# Affect fluctuations examined with ecological momentary assessment in patients with current or remitted depression and anxiety disorders

**DOI:** 10.1017/S0033291720000689

**Published:** 2021-08

**Authors:** R. A. Schoevers, C. D. van Borkulo, F. Lamers, M.N. Servaas, J. A. Bastiaansen, A. T. F. Beekman, A. M. van Hemert, J. H. Smit, B. W. J. H. Penninx, H. Riese

**Affiliations:** 1 University of Groningen, University Medical Center Groningen, Department of Psychiatry, Interdisciplinary Center for Psychopathology and Emotion regulation, Groningen, The Netherlands; 2 University of Amsterdam, Department of Psychological Methods, Amsterdam, The Netherlands; 3 Amsterdam UMC, Vrije Universiteit, Department of Psychiatry, Amsterdam Public Health research institute, Amsterdam, The Netherlands; 4 Department of Education and Research, Friesland Mental Health Care Services, Leeuwarden, The Netherlands; 5 Department of Psychiatry, Leiden University Medical Center, Leiden, The Netherlands

**Keywords:** Affect variability, anxiety disorder, depressive disorder, ecological momentary assessment

## Abstract

**Background:**

There is increasing interest in day-to-day affect fluctuations of patients with depressive and anxiety disorders. Few studies have compared repeated assessments of positive affect (PA) and negative affect (NA) across diagnostic groups, and fluctuation patterns were not uniformly defined. The aim of this study is to compare affect fluctuations in patients with a current episode of depressive or anxiety disorder, in remitted patients and in controls, using affect instability as a core concept but also describing other measures of variability and adjusting for possible confounders.

**Methods:**

Ecological momentary assessment (EMA) data were obtained from 365 participants of the Netherlands Study of Depression and Anxiety with current (*n* = 95), remitted (*n* = 178) or no (*n* = 92) DSM-IV defined depression/anxiety disorder. For 2 weeks, five times per day, participants filled-out items on PA and NA. Affect instability was calculated as the root mean square of successive differences (RMSSD). Tests on group differences in RMSSD, within-person variance, and autocorrelation were performed, controlling for mean affect levels.

**Results:**

Current depression/anxiety patients had the highest affect instability in both PA and NA, followed by remitters and then controls. Instability differences between groups remained significant when controlling for mean affect levels, but differences between current and remitted were no longer significant.

**Conclusions:**

Patients with a current disorder have higher instability of NA and PA than remitted patients and controls. Especially with regard to NA, this could be interpreted as patients with a current disorder being more sensitive to internal and external stressors and having suboptimal affect regulation.

## Introduction

Depressive and anxiety disorders as they are currently classified (DSM diagnosis) are heterogeneous in terms of symptomatology (Fried, 2017), underlying pathophysiological mechanisms, and clinical course (Jentsch et al., 2015; Verduijn et al., 2017). Moreover, depressive and anxiety disorders often co-occur (Kessler et al., 2003) and patients with different diagnoses may show overlapping symptom profiles (Wardenaar & de Jonge, 2013). This lack of diagnostic specificity likely plays a role in the overall modest efficacy of current psychological and pharmacological treatments (Driessen, Hollon, Bockting, Cuijpers, & Turner, 2015; Turner, Matthews, Linardatos, Tell, & Rosenthal, 2008).

To add to this complexity, symptoms that patients experience may show considerable variation over time (Ben-Zeev & Young, 2010; Thompson et al., 2012). Such fluctuations are not represented in regular psychiatric diagnoses that are based on patients' retrospective accounts of their overall symptomatology in the weeks or months preceding a diagnostic interview, and the clinical presentation at that moment. Momentary affect, described as the ‘quick-moving reactions that occur when organisms encounter meaningful stimuli that call for adaptive responses’ (Rottenberg, 2005), can reliably be captured with ecological momentary assessment (EMA) (Stone & Shiffman, 1994; Trull & Ebner-Priemer, 2009). With its short but multiple assessments, EMA is useful to monitor frequency and fluctuations (i.e. dynamics) of momentary affect in normal life over a period of several days or weeks (aan het Rot, Hogenelst, & Schoevers, 2012).

**Fig. 1.**
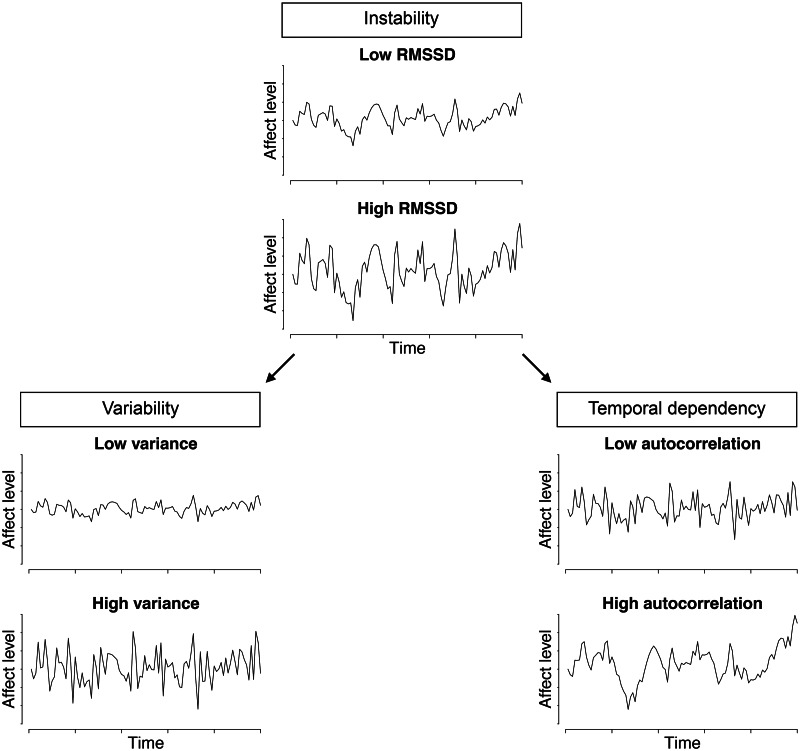
Illustration of the three components of affect fluctuation patterns: instability, variability, and temporal dependency. Instability (quantified by the RMSSD; top panels for illustrations of patterns with low and high RMSDD) has two components: variability (quantified by the variance; bottom left panels for patterns with low and high variance) and temporal dependency (quantified by autocorrelation; bottom right panels for patterns with low and high autocorrelation). Adapted from Houben et al. (2015), data were simulated according to Jahng et al. (2008).

It has been suggested that affect fluctuations can be a useful marker of emotional dysregulation, as a characteristic of depressive and anxiety disorders (Shackman et al., 2016; Trull, Lane, Koval, & Ebner-Priemer, 2015). Affect fluctuations are composed of two components: variability and temporal dependency (see Fig. 1; Houben, Van Den Noortgate, and Kuppens, 2015; Jahng, Wood, and Trull, 2008). Variability entails the extremity of affect levels that a person experiences and is typically quantified by the within-person variance (WPV).

Temporal dependency, the second component of affect fluctuations, describes how affect changes over time. This is quantified by the autocorrelation. When affect changes slowly, the autocorrelation will be high, indicating that the current affect state can be well predicted from the previous moment. Phrased differently, when the autocorrelation is high, there is a resistance to change of affect. This phenomenon of reduced reactivity of affect has been called *inertia*, which is found to be associated with psychological maladjustment (Bylsma, Morris, & Rottenberg, 2008; Kuppens, Allen, & Sheeber, 2010).

To study affect fluctuations, both components of fluctuations should be taken into account. This can be accomplished by investigating *instability*: changes from one moment to the next, thereby capturing both variability and temporal dependency. Instability can be quantified by the root mean square of successive differences (RMSSD) of affect time-series data, which typically capture these two components of affect fluctuations (Jahng et al., 2008).

Earlier studies on affect fluctuations showed, for example, that depressed patients had higher variability in negative affect (NA) than controls (Peeters, Berkhof, Delespaul, Rottenberg, & Nicolson, 2006), but also in positive affect (PA; Gilbert, 2012; Gruber, Kogan, Mennin, and Murray, 2013; Peeters et al., 2006), and a greater resistance to change of affect (Kuppens et al., 2010). An earlier EMA study found that patients with remitted depression showed increased affect instability in NA compared to healthy controls, and no group differences for PA (Servaas et al., 2017). Greater variability and instability were observed for sad mood in MDD and anxious mood in different mood/anxiety diagnostic groups in a study by Lamers et al. (2018), but group differences were not observed for autocorrelation of sad or anxious moods. A meta-analysis has shown that overall, low psychological well-being co-occurs with more variable, unstable and more inert emotions, with more pronounced results for negative compared to positive emotions (Houben et al., 2015).This could be indicative of increased vulnerability for developing another depressive episode either as a result of an earlier episode or as a more general vulnerability trait (aan het Rot et al., 2012).

For a better understanding of depression and anxiety disorders and its treatment, it is highly relevant to study patients' individual affect fluctuations (aan het Rot et al., 2012). A first question of interest is whether such individual patterns are indicative of clinical group membership, for example in subjects with a current disorder, remitted disorder, and healthy controls. To investigate this, studies are needed that combine thorough diagnostic assessments and follow-up of a large sample of patients and controls with systematic monitoring of affect using EMA. The Netherlands Study of Depression and Anxiety (NESDA; Penninx et al., 2008) now offers the unique possibility to zoom in on daily affect dynamics of the participants using 2 weeks of intensive EMA measurements of PA and NA. In the current study, we investigated whether participants in different diagnostic groups (i.e. with no, remitted, or current depressive and/or anxiety disorder) are characterized by different affect fluctuations.

Our main hypothesis is that affect instability (RMSSD) of both NA and PA is higher in participants with a current episode of depressive or anxiety disorder, followed by remitted patients and lowest in the controls. Second, when excluding participants with pure anxiety to make our sample more similar to that in previous studies that focused on depression, we expect a similar pattern in affect instability across groups. As WPV and autocorrelation have been used in other studies as measures for investigating fluctuation patterns (Koval et al., 2013; Trull et al., 2015), we will also investigate these in our three groups and investigate the correlations between all fluctuation measures.

## Methods

### Sample

Participants of the EMA & Actigraphy sub-study (NESDA-EMAA) were selected from the fifth wave of the NESDA study. NESDA is an ongoing longitudinal cohort study aimed at examining the long-term course of depressive and anxiety disorders in different health care settings and phases of illness. Full details about NESDA are given elsewhere (Penninx et al., 2008). In short, 2981 participants were initially included at baseline assessment in 2004–2007, and from these 1776 participated in the fifth wave at the 9-year regular follow-up assessment (2014–2017) for a regular face-to-face interview, including a psychiatric diagnostic interview (details below). At this fifth wave, siblings of a subsample of NESDA participants were also invited to participate if they had the same biological parents, and if their related NESDA participant had a depressive or anxiety disorder at any of the NESDA waves and participated in at least two of the four previous waves as well as in the current regular interview. The NESDA study, including the EMAA component, was approved by the VUmc ethical committee (reference number 2003/183) and all participants gave informed consent for both the regular interview and the EMAA component.

### Participants and procedures NESDA-EMAA sub study

#### Enrolment

**Fig. 2.**
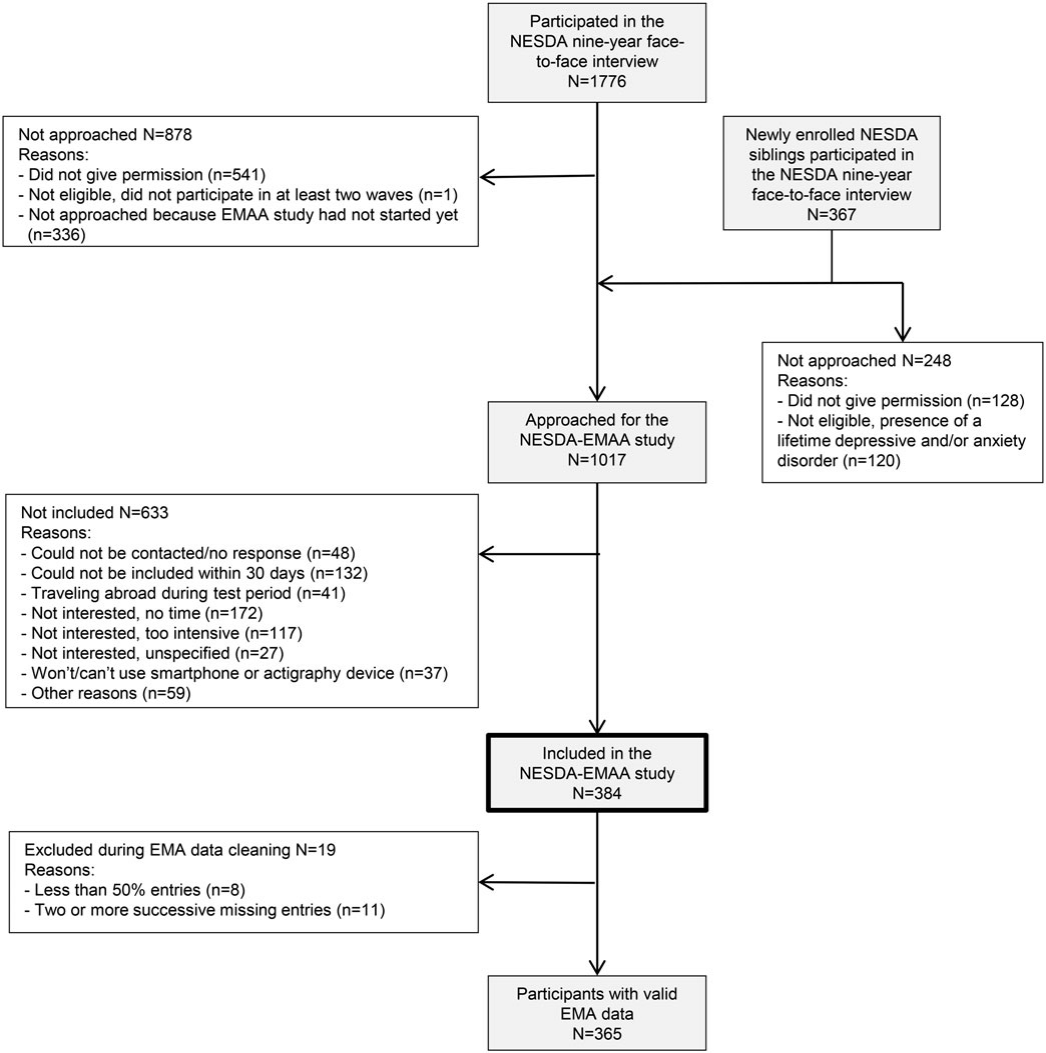
Flowchart of the enrollment and inclusion of the participants of the NESDA-EMAA study (see Methods section for details).

A flowchart depicting the enrolment and inclusion criteria is given in Fig. 2. After the 9-year interview, participants of NESDA who were eligible and willing to participate in the NESDA-EMAA sub-study were invited to one of the four research facilities within 1 month. For this study we invited NESDA participants who: participated in at least two of the previous NESDA waves, consented to be approached for this sub-study, participated in the regular interview ⩽31 days prior to starting the EMA measurements, had good mastery of the Dutch language, were familiar with smartphone use and willing to wear a wrist-worn actigraphy device. Siblings were invited if they did not have a current or past diagnosis of a depressive and/or anxiety disorder or another severe psychiatric disorder (such as psychotic or severe addiction disorder). In total, 384 participants were fully informed and given time to ask questions prior to participation.

#### Intake

Participants received instructions on the EMA assessments, that is an electronic diary (details below) and a wrist-worn actigraphy device (details in Difrancesco et al., 2019), plus a written user instruction for the EMA assessments on their personal smartphone from trained research assistants. Research assistants were trained during a 1-day workshop and remained in regular contact with a central coordinator to run the experiment conform the research protocol. In case participants did not possess a smartphone, or their phone was not suitable for participation (e.g. no internet bundle), a smartphone was provided for the duration of the study (*n* = 107, 27.9%). Data were gathered with our secured server system (RoQua, Sytema and Van der Krieke, 2013). The system was programmed to send text messages with links to online questionnaires to the respondent's smartphone. Timing of assessments was personalized to fit participant's natural wake-sleep rhythm during the week and weekend; the first daily assessment was aimed to be 1–2 h after waking-up in the morning.

#### Ecological momentary assessment

Participants took part in the EMA for 2 weeks and completed a set of items 5 times a day (i.e. every 3 hours; fixed design). Participants were instructed to complete the questionnaires as soon as possible after receiving the text message (beep), preferably within 15 min, but at least within 60 min. Patients received a reminder after 30 min. Participants were shown an example of a personalized feedback report and told that they could obtain such a report if they filled-out at least 80% of all assessments. In this report, graphs and explanatory text were given for a participant that reported affect, sleep duration, life style factors and the relationship between mood and sleep. During the assessment period participants could call/text the research assistants if they had questions. According to the standard procedure, research assistants called the participants twice: 24 h and 1 week after they had started the EMA to ask whether they had questions, and to motivate them to continue to fill-out the electronic diaries. Every other day, the research assistants monitored progress of the respondent in the RoQua system and called the respondent when implausible response patterns were given or when more than three subsequent assessments were missed, to find out whether there was a problem. Participants were reimbursed €20 and received a personalized report of their EMA assessments afterwards.

### Measures

#### Diagnosis of depression and/or anxiety disorders

Similar to the previous waves, at the 9-year follow-up, DSM-IV diagnoses of depressive disorders (dysthymia and major depressive disorder) and anxiety disorders (social anxiety disorder, panic disorder with and without agoraphobia, agoraphobia and generalized anxiety disorder) were established with the Composite International Diagnostic Interview (CIDI, version 2.1; Wittchen, 1994). The interviews were conducted by trained clinical research staff.

The 384 participants included in this study were divided into three groups based on their CIDI diagnosis, analogous to Difrancesco et al. (2019): (1) a current group with a depressive and/or anxiety disorder in the past 6 months at the 9-year follow-up interview (*n* = 100), (2) a remitted group with a lifetime depressive disorder and/or anxiety disorder but not in the 6 months prior to the 9-year follow-up interview (*n* = 190), and (3) a control group with no lifetime history of depressive and/or anxiety disorders (*n* = 94).

#### Daily EMA questionnaires

The questionnaires that were assessed five times a day had up to 31 items per time point, and contained both momentary affect state items and other items on activities, context or lifestyle. To assess momentary affect states we included items of the study Uncovering the Positive Potential of Emotional Reactivity study (Bennik, 2015), which balance emotional adjectives on both the (positive and negative) valence dimension and (high/low) arousal dimension of emotional experience (Watson & Tellegen, 1985). The items covered high and low arousal, positive and negative momentary affect states: I feel satisfied, relaxed, upset, cheerful, irritated, listless, down, energetic, enthusiastic, nervous, bored, calm, and anxious. They were rated on a 7-point Likert scale ranging from ‘1 = not at all’ to ‘7 = very much’. A PA subscale was calculated by averaging the PA items (items: at this moment I feel satisfied, relaxed, cheerful, energetic, enthusiastic, and calm), and a NA subscale was calculated by averaging the NA items (items: at this moment I feel upset, irritated, listless/apathic, down, nervous, bored, anxious). Immediately after the last daily EMA questionnaire, participants received a text message with a link to an addendum questionnaire. This questionnaire contained 17 items to obtain information on, for example, changes in medication use and evaluations of the NESDA-EMAA study. These items were not used in the current paper.

### Statistical analysis

#### Data cleaning

During the daily EMA assessments, in total 24 537 observations of 384 participants were received (i.e. on average 63.90 observations per participant, s.d. = 8.88). Of all sent EMA assessments to all participants, only 8.72% were missing. Data cleaning steps, which included handling missing data, are described in detail in online Supplemental materials S1. With the resulting exclusion of 19 participants due to too much incomplete data, 365 participants were used for our analyses (see Fig. 2) with *M* = 65.38 (s.d. = 4.25) valid responses.

#### Clinical characteristics

To describe our EMA sample, the following characteristics were used: severity of depressive and anxiety symptoms, number of comorbid psychiatric disorders, duration of depressive or anxiety disorders, age of onset, and medication use (i.e. antidepressant and benzodiazepines use). Severity of depressive and anxiety symptoms were assessed with the 30-item Inventory of Depressive Symptomatology (IDS; Rush, Gullion, Basco, Jarrett, and Trivedi, 1996) and the Beck Anxiety Inventory (BAI; Beck, Epstein, Brown, and Steer, 1988). Analogous to Difrancesco et al. (2019), the number of comorbid psychiatric disorders was the sum of current depressive and anxiety diagnoses at 9-year follow-up. Frequency of depressive or anxiety disorders was calculated as a count of the number of waves at which patients reported a depression and/or anxiety diagnosis during the in-between follow-up periods (ranging from 1–5 waves). Age of onset was derived from the CIDI. Antidepressant and benzodiazepine use was based on drug container inspection, and medications were coded according to the World Health Organization Anatomical Therapeutic Chemical (ATC) classification. Antidepressant and benzodiazepine use was considered present if participants reported using it more than 50% of days in a month. We included selective serotonin reuptake inhibitors (SSRIs, ATC code N06AB), tricyclic antidepressant (TCA, ATC code N06AA) and other antidepressants (ATC codes N06AF, N06AG, N06AX); benzodiazepines included ATC codes N03AE, N05BA, N05CD, and N05CF.

#### The RMSSD

Fluctuation patterns over time in PA and NA were assessed as the RMSSD (Houben et al., 2015; Jahng et al., 2008; Servaas et al., 2017) between scores on single items. The RMSSD is measured as the square root of the average of the squared differences between affect at measurement *i* and *i* + 1. For *N* measurements, the RMSSD is described as:
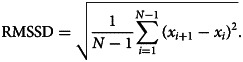
This measure quantifies affect instability by capturing both variability and temporal dependency in EMA data (Houben et al., 2015; Jahng et al., 2008). WPV (measuring variability) and autocorrelation (measuring temporal dependency) are described in online Supplement S4.

#### Statistical testing

Person-mean levels of PA and NA, and RMSSD of PA and NA (RMSSD_PA and RMSSD_NA) subscale scores were compared between groups with no, remitted, and current depressive disorders. All measures were not normally distributed according to the Shapiro–Wilk test. Also, according to Levene's test, variances of our measures were not homogeneous. This means that, since the assumption of normality was violated, we could not use parametric tests (e.g. ANOVA). Therefore, we used the non-parametric Kruskal–Wallis test as an omnibus test and used epsilon square (*ɛ***^2^**) as the effect size. Since *ɛ***^2^** is a squared variable that runs from 0 to 1, we use the same interpretation for as a correlation coefficient (Rea & Parker, 1992). Squaring the upper and lower bounds of each bin provides the following interpretations: effect sizes up till 0.04 are considered as weak, up till 0.16 as moderate, up till 0.36 as relatively strong, up till 0.64 as strong and from 0.64 onwards as very strong.

The non-parametric Dunn's test was used for *post-hoc* pairwise comparisons with a Bonferroni correction for multiple testing (*α* = 0.05). Non-parametric tests have lower power than their parametric counterparts, which means that they err on the side of caution. Correlations between RMSSD of PA and of NA were explored with Spearman's correlations.

To align more with previous research that focused on patients with depression, either with or without comorbid anxiety, the analysis was repeated after excluding those with pure (current and remitted) anxiety disorder. We defined pure current anxiety as having an anxiety disorder at 9-year follow-up and no depressive disorder at any of the waves. Similarly, pure remitted anxiety disorder is defined as having a remitted anxiety status at 9-year follow-up and no depressive disorder at any of the waves.

#### Sensitivity analyses

An important potential confounder is the level of affect. Variability can be confounded by the level of affect since measurements of affect are bounded by the rating scale of the items. Healthy individuals, for example, are typically known to show low levels of NA and measurements are, therefore, highly skewed. This inherently means that variability will also be low. To correct for such a floor effect we additionally used the corrected RMSSD (cRMSSD; van Roon, Snieder, Lefrandt, de Geus, and Riese, 2016):
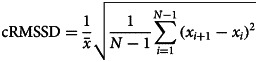
in which 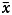 is the mean of all *x*_*i*_.

For all tests, we used significance level *α* = 0.05 (unless stated otherwise) and *p* values were Bonferroni corrected for *post-hoc* comparisons. All analyses were performed with the statistical software R (version 3.4.3), or SPSS (version 24).

## Results

### Sample descriptives and correlations

Demographics and clinical characteristics of our sample are given in Table 1. Of the total sample (*n* = 365), 95 had a current, 178 had a remitted, and 92 had no current or remitted depressive and/or anxiety disorder (control). The group with a current disorder consisted of 28.4% who had only had a depressive disorder, 40% only an anxiety disorder, and 31.6% with combined (comorbid) disorders. For the remitted group: 26.1% of the participants had a remitted depressive disorder, 13.6% had remitted anxiety disorders, and 60.2% had combined (comorbid) remitted disorders. The three groups differed significantly in depressive and anxiety symptom scores with the current group scoring the higher than remitters, who scored higher than controls (*p's* < 0.001). Also, participants in the current group used antidepressants more frequently than the remitted group, who used antidepressants more frequently than controls (*p's* < 0.001).

**Table 1.** Demographic, psychiatric, psychological characteristics and medication use in our NESDA sample (*n* = 365)

	Current depressive and/or anxiety disorders	Remitted depressive and/or anxiety disorders	No depressive and/or anxiety disorders	*p* value
*N*	95	178	92	
Demographics				
Age, mean (s.d.)^a^	49.6 (11.4)	48.4 (13.1)	51.2 (12.8)	0.41
Female, *n* (%)^b^	59 (62.1)	125 (70.2)	51 (55.4)	0.048
Years of education (years), mean (s.d.)	12.5 (3.4)	12.7 (2.8)	14.0 (2.8)	0.005
Psychopathology				
Only depressive disorders, *n* (%)	27 (28.4)	46 (26.1)	-	
Only anxiety disorders, *n* (%)	38 (40.0)	24 (13.6)	-	
Depressive & anxiety disorders, *n* (%)	30 (31.6)	106 (60.2)	-	
Number of psychiatric disorders, median (IQR)	1 (1 - 2)	-	-	
Frequency of psychiatric disorders (number of waves), median (IQR)	5 (4 - 5)	-	-	
Age of depressive disorder or anxiety onset, mean (s.d.)	16.8 (11.9)	-	-	
Psychological scales				
IDS, mean (s.d.)^a^	24.9 (12.7)	12.6 (8.5)	5.5 (3.8)	<0.001
BAI, mean (s.d.)^a^	13.3 (9.3)	5.9 (5.6)	1.6 (1.9)	<0.001
Medication use				
Antidepressant users, *n* (%)^b^	34 (35.8)	36 (20.2)	2 (2.2)	<0.001
Benzodiazepines users, *n* (%)^b^	5 (5.3)	8 (4.5)	0 (0.0)	0.10

s.d., standard deviation; IQR, interquartile range; IDS, Inventory of Depressive Symptomatology: BAI, Beck Anxiety Inventory.

a Analysis of variance (ANOVA).

b χ^2^ test.

The current group had significantly lower levels of person-mean PA and significantly higher levels of person-mean NA than the remitted group, which in turn had significantly lower levels of PA and significantly higher levels of NA than the control group (Table 2; and online Supplemental Table S2 for the accompanying information on the item level and Table S3 for correlations on the item level and the PA and NA scales). The correlation between PA and NA was strongest in the group with a current depressive disorder (−0.75) and weakest in the healthy control group (−0.59), with the remitters in between the two (−0.70, Table 2).

**Table 2.** Descriptives (mean, standard deviation, skewness, kurtosis, and correlation) of PA and NA scales

	Current (*n* = 95)				Remitted (*n* = 178)				Control (*n* = 92)			
	Mean	s.d.	Skew	Kurtosis	Mean	s.d.	Skew	Kurtosis	Mean	s.d.	Skew	Kurtosis
PA^a^	4.17	0.67	−0.41	0.24	4.84	0.75	−0.63	0.78	5.41	0.66	−0.90	2.11
NA^a^	2.15	0.77	0.98	1.72	1.51	0.54	1.86	5.28	1.17	0.25	2.87	11.38
r(PA,NA)	−0.75				−0.70				−0.59			

PA, positive affect; NA, negative affect.

a Significant difference in means across all groups (Kruskal–Wallis test and *post-hoc* comparisons all *p* < 0.00001).

### Differences in RMSSD across diagnostic groups

**Fig. 3.**
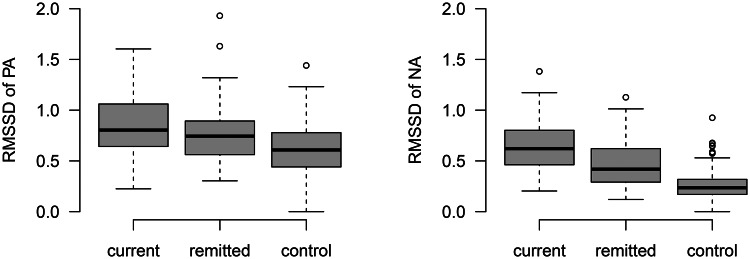
Boxplots of person-mean RMSSD of PA and NA subscales of the diagnostic groups (see method section for details).

We found a statistically significant difference in both RMSSD_PA and RMSSD_NA between the diagnostic groups [H(2) = 31.97, *p* = <0.0001 and H(2) = 112.92, *p* < 0.0001, respectively]. These differences were moderate for RMSSD_PA (*ɛ*^2^ = 0.09) and relatively strong for RMSSD_NA (*ɛ*^2^ = 0.31). *Post-hoc* comparisons revealed that the current group had significantly higher median RMSSD of PA and NA (0.80 and 0.62, respectively) than the remitted group (0.74 and 0.42, respectively), who had a significantly higher median than controls (0.61, 0.24; see Table 3 and Fig. 3). The interquartile range (see Table 3) was highest for the RMSSD_PA of the current group (0.64–1.08) and lowest for the RMSSD_NA of controls (0.17–0.32).

**Table 3.** Median (IQR) RMSSD of PA and NA in diagnostic groups

RMSSD	Current (*n* = 95)	Remitted (*n* = 178)	Control (*n* = 92)	*p* value^a^	*ɛ*^2^
PA	0.80 (0.64–1.08)	0.74 (0.56–0.89)	0.61 (0.44–0.78)	<0.0001^b^	0.09
NA	0.62 (0.46–0.80)	0.42 (0.29–0.62)	0.24 (0.17–0.32)	<0.0001^b^	0.31

RMSSD, root mean square of successive differences; IQR, interquartile range; PA, positive affect; NA, negative affect; ***ɛ*^2^**, effect size.

a Kruskal–Wallis test.

b Dunn's test, *p* < 0.05 for all three comparisons, Bonferroni corrected.

In a reanalysis, we excluded patients with a current (*n* = 7) or remitted pure anxiety disorder (*n* = 25), resulting in a sample of 333 participants^1^. In this reanalysis, comparisons of RMSSD of all diagnostic groups were again statistically significant, except for the difference in RMSSD-PA between the current and remitted group, which was no longer statistically significant. The significance of the other comparisons however remained (see online Supplemental Table S6). Effect sizes of the differences in RMSSD_PA and RMSSD_NA without patients with pure anxiety were, however, similar to the original effect sizes (*ɛ*^2^ = 0.10 and *ɛ*^2^ = 0.34, respectively), indicating that the non-significant difference of RMSSD_PA between the current and remitted group might be due to the loss of power because sample size was reduced by excluding patients with pure anxiety.

Besides the RMSSD, we also inspected differences between the groups on the separate components of RMSSD: WPV and autocorrelation. Similar differences were found for these components of RMSSD except that the current and remitted group did not differ significantly with respect to autocorrelation of PA [Tables S4.2 and S4.3 for median (IQR) of WPV and autocorrelation per diagnostic group, and Figs S4.1 and S4.2 for the accompanying boxplots]. Interestingly, the RMSSD and WPV were very strongly correlated (PA: *r* = 0.92, NA: *r* = 0.97). The correlation between the autocorrelation and RMSSD was, however, absent with respect to PA (*r* = −0.02) and moderate with respect to NA (*r* = 0.37). WPV and autocorrelation were moderately correlated (PA: *r* = 0.30, NA: *r* = 0.55; see online Supplemental Table S4.1 for correlations between all three variability measures).

### Sensitivity analyses

An important potential confounder for our results was that mean levels of affect could differ across groups. Repeating previous analyses with the cRMSSD (the mean corrected RMSSD) showed that, similar to analysis of the uncorrected RMSSD, the omnibus test revealed significant differences for both cRMSSD_PA and cRMSSD_NA (see online Supplemental Table S5). *Post-hoc* tests revealed that, with respect to cRMSSD_PA, all groups still differed significantly. With respect to cRMSSD_NA, the differences between current and control, and between remitted and control remained significant, but the difference between current and remitted did not. Although cRMSSD_NA of the current group was still higher than that of the remitted group, this difference was no longer statistically significant (see online Supplemental Table S5). Effect sizes of differences of cRMSSD_PA and cRMSSD_NA were relatively strong (*ɛ*^2^ = 0.19 and *ɛ*^2^ = 0.18, respectively).

## Discussion

The aim of this paper was to describe affect fluctuations, and specifically affect instability of PA and NA, in a large and well-phenotyped cohort of patients with depressive and anxiety disorders and controls. We examined whether these patterns of affect fluctuations are different for patients who are in a current episode, remitted patients and healthy controls. Our results showed that these three groups differed significantly on the RMSSD (and WPV) of both PA and NA. The most consistent differences were found between patients and controls, after correcting for mean PA and NA levels, when excluding patients with pure anxiety disorder and when regarding autocorrelation. Results had moderate to relatively strong effect sizes.

Our study is one of the largest to date, with EMA data from nearly 400 participants whose disease course has been monitored for 12 years prior to EMA assessment. Despite the extra effort that was required by filling-out the daily EMA questions five times per day for 2 weeks, participants were interested to participate and there were very little missing data. This shows that adding a more fine-grained measure of momentary affect to studies such as NESDA is acceptable and feasible, both for patients in an episode, in remission and for controls.

Importantly, our data show that affect fluctuations indeed differ between the three groups. Moreover, groups also differed with respect to the two separate components: variance and temporal dependency. In clinical terms, this implies that patients with a current disorder not only have high levels of NA and low levels of PA, but also differ on their affect dynamics: that is, a higher level of instability and higher autocorrelation (inertia) of affect. This may be interpreted as, especially with regard to NA, that patients with a current disorder have larger but slower changes in affect (Koval, Pe, Meers, & Kuppens, 2013). This mechanism has been attributed to being more sensitive to internal and external stressors (large affect instability) in combination with having suboptimal affect regulation (slow change in affect) (Myin-Germeys et al., 2003; Wichers et al., 2007). Although it should be noted that the current results are based on group level analyses, they appear to be in line with clinical observations in individual patients. Depressed patients tend to be more sensitive to their daily context and/or are known to be rather persistent in their sad mood by interpreting negative, positive and even ambiguous events, in a negative way. However, whether intervention selection based on the different affect fluctuation indices, preferably calculated from personalized EMA assessments, is feasible awaits further empirical study (Koval et al., 2013).

In addition to most previous studies that have not looked at this issue, we have also shown differences between remitted patients and healthy controls. These may reflect either an inherent vulnerability to develop depression or anxiety disorder, or a consequence of an earlier episode. According to the set-point theory (Ormel, Von Korff, Jeronimus, & Riese, 2017), changes in the affect regulation system may be reflected in lasting set-point changes (higher for NA, lower for PA) with increased instability of PA en NA leading to higher vulnerability for relapse after remission. Further longitudinal studies are needed to investigate this. Additionally, the underlying mechanism can be studied by determining whether, when patients recover, their affect response patterns, and capability of maintaining homeostasis improve when confronted with daily life stress (aan het Rot et al., 2012), and how this relates to patients in an episode or healthy controls. This will be explored in a future study with NESDA-EMAA data.

Moreover, it would be of great interest to determine if and how treatment could impact on the affect fluctuations we found. An earlier study, in patients who were treated with antidepressants, showed that PA increased more in reaction to positive events and NA decreased less after negative events but this study was not able to determine whether these effects were specific to the form of treatment they received (Wichers et al., 2009). Adding EMA to randomized controlled trials would be an interesting way to reveal underlying mechanisms that may explain such more subtle treatment effects. Furthermore, as reviewed by Bos, Schoevers, & aan het Rot (2015) studies have also used EMA patterns before treatment to predict outcome, to detect vulnerability for relapse, and to determine how long and in what contexts antidepressant medication is helpful for patients. It should be noted that NESDA is a naturalistic longitudinal cohort in which treatment was in no way controlled or standardized.

The field of EMA studies is rapidly evolving and several measures of fluctuation have been studied. For comparison with these studies, and to determine how different measures would relate to the outcomes of our study, we have also looked at WPV and autocorrelation and their correlation with RMSSD. We found that WPV was very strongly correlated to RMSSD while the correlation between autocorrelation and RMSSD was absent for PA and moderate for NA. Apparently, the RMSSD of PA, which is composed of both variance and autocorrelation, is exclusively driven by the variance and not by the autocorrelation. When interpreting these results, a number of limitations need to be considered. First, the analyses in the present paper were not performed using multilevel modeling because the RMSSD is calculated at the person level and not at the assessment level. Therefore, we investigated differences across groups in a two-step procedure: we calculated RMSSD at person level first, and then investigated differences in median across groups, thereby ignoring measurement error. With a multi-level approach, differences between groups could be investigated in one step, thereby taking measurement error into account. The two-step procedure is, however, in line with other literature using RMSSD or related measures (Heininga, van Roekel, Wichers, & Oldehinkel, 2017; Thompson et al., 2012). Second, future research is needed to determine what fluctuation measures are best able to distinguish between diagnostic groups. Third, differences in RMSSD of affect can be confounded by group differences in s.d. or mean levels of affect (Bos, de Jonge, & Cox, 2018). We accounted for differences in s.d. by using a statistical test that is robust for such characteristics. As discussed above, controlling for mean differences affected the comparison of NA variability between the current and remitted group that was no longer statistically different, but not the other comparisons.

In sum, in the current study we showed both PA and NA fluctuated more in participants with a current depression compared to controls, even after controlling for differences in mean levels of PA and NA. This suggests that EMA assessment is able to pick up relevant aspects of more subtle affect dynamics that are part of the diseased state, as well as possible vulnerability characteristics in healthy or remitted participants. It is tempting to speculate that findings of this study are promising as a starting point for research exploring more differentiated clinical phenotypes and affect fluctuation patterns. These patterns may be relevant as indicators of vulnerability as well as markers that may be used to guide intervention in clinical practice.

## Footnotes

1 The number of pure (current and remitted) anxiety deviate from the numbers in Table 1 due to a different definition of pure anxiety (described in the Methods section). For example, for the number of patients with only a current anxiety disorder in Table 1, previous waves were not taken into account.

## Supplementary material

For supplementary material accompanying this paper visit https://doi.org/10.1017/S0033291720000689. click here to view supplementary material
