# Supplementary material for: Affect fluctuations examined with ecological momentary assessment in patients with current or remitted depression and anxiety disorders
Source: Psychol Med. 2020 Apr 1;51(11):1906–15. doi: 10.1017/S0033291720000689 (PMC8381239; doi:10.1017/S0033291720000689)
Supplement: Supplementary file 1 [file S0033291720000689sup001.docx]

**Supplementary Materials**

**Affect fluctuations examined with ecological momentary assessment in patients with current or remitted depression and anxiety disorders**

R.A. Schoevers*, C.D. van Borkulo*, F. Lamers, M.N. Servaas, Bastiaansen, A.T.F. Beekman, A.M. van Hemert, J. Smit, B.W.J.H. Penninx, H. Riese

*****contributed equally to this manuscript

**Supplementary Methods and Results**

Contents

[S1 EMA data cleaning 2](#_Toc14344467)

[S2 Mean (SD) of the person-means of the affect items used for calculations of the positive and negative subscales. 4](#_Toc14344468)

[S3 Correlations between the person-mean affect items and the positive and negative affect sum scores 5](#_Toc14344469)

[S4 Explanation of within-person variance and autocorrelation, its correlation with RMSSD, and median (IQR) per group 6](#_Toc14344470)

[S5 Median (IQR) of the corrected RMSSD (cRMSSD) of positive and negative affect in diagnostic groups 9](#_Toc14344471)

[S6 Median (IQR) RMSSD of positive and negative affect in diagnostic groups after excluding respondents with pure current or pure remitted anxiety disorder 10](#_Toc14344472)

[References 11](#_Toc14344473)

# S1 EMA data cleaning

During the daily EMA assessments, in total 24537 observations of 384 participants were assessed (i.e., on average 63.90 observations per participant, SD = 8.88). As outlined in the Method section, respondents were shown an example of a personalized feedback report and told that they could obtain such a report if they would fill out at least 80% of all assessments. In total 93% (n=357) had been able to fill-out 80% of all assessments. Four of the 384 respondents who were invited to fill out the addendum questionnaire did not do so. However, as only the affect items (item 3-15, S2 Tables) were used for the current manuscript, data cleaning was proceeded with these four respondents included.

Several checks were performed on the EMA item data prior using these in further statistical analyses. First, we checked whether responses exceeded the minimum or maximum score (<1 or >7) (0 observations, 0 participants). Second, exceedance of the maximum response time was checked. Respondents were instructed to fill-out the questionnaires as soon as possible after receiving the text message (beep), preferably within 15 minutes but no later than 60 minutes. The patient received a reminder after 30 minutes. We excluded observations that were not uploaded 65 minutes after the participants were invited by a text message to fill-out the questionnaire (18 observations, from 17 participants). On average respondent filled out the questionnaire 12:44 minutes (sd = 14:30) after the beep. Measurements took on average 3:06 minutes (sd = 2:28) to complete. On average measurements started at 8:57:32 (sd = 1:04:13). Third, we identified missing data, that is, observations that are missing because individuals opened the questionnaire but did not fill it in (0 observations, 0 participants) or because of technical failure (e.g server down n=19). Fourth, participants with a response rate below 50% (35 of 70 observations; in line with Servaas et al. (2017)) were excluded from the analyses (*n* = 8)). Fifth, to prevent successive missing data from influencing the RMSSD, we also excluded participants with more than one series of two or more successive missings (*n* = 11). With the resulting exclusion of 19 participants, 365 participants were used for our analyses (see Figure 1) with on average 65.38 (sd = 4.25) valid responses.

# S2 Mean (SD) of the person-means of the affect items used for calculations of the positive and negative subscales.

|  | **Current (*n* = 95)** | **Remitted (*n* = 178)** | **Control (*n* = 92)** | ***p***^a^ |
| --- | --- | --- | --- | --- |
| **satisfied** | 4.49 (0.78) | 5.18 (0.77) | 5.67 (0.71) | **<0.0001** |
| **relaxed** | 4.32 (0.69) | 4.96 (0.72) | 5.54 (0.64) | **<0.0001** |
| **cheerful** | 4.21 (0.76) | 4.88 (0.84) | 5.42 (0.74) | **<0.0001** |
| **energetic** | 3.79 (0.78) | 4.50 (0.90) | 5.15 (0.82) | **<0.0001** |
| **enthusiastic** | 3.85 (0.86) | 4.59 (0.95) | 5.15 (0.85) | **<0.0001** |
| **calm** | 4.35 (0.75) | 4.95 (0.76) | 5.54 (0.62) | **<0.0001** |
| **upset** | 1.69 (0.77) | 1.31 (0.50) | 1.09 (0.19) | **<0.0001** |
| **irritated** | 2.21 (0.87) | 1.63 (0.58) | 1.30 (0.39) | **<0.0001** |
| **listless** | 2.65 (1.06) | 1.79 (0.86) | 1.32 (0.47) | **<0.0001** |
| **down** | 2.56 (1.22) | 1.58 (0.82) | 1.14 (0.32) | **<0.0001** |
| **nervous** | 2.36 (1.02) | 1.63 (0.78) | 1.18 (0.37) | **<0.0001** |
| **bored** | 1.84 (0.90) | 1.34 (0.52) | 1.15 (0.43) | **<0.0001** |
| **anxious** | 1.75 (0.89) | 1.28 (0.64) | 1.04 (0.14) | **<0.0001** |

^a^ *p*-value applies to both Kruskal–Wallis omnibus test and Dunn’s test for all Bonferroni corrected comparisons

# S3 Correlations between the person-mean affect items and the positive and negative affect sum scores

|  | satisfied | relaxed | cheerful | energetic | enthusiastic | calm | upset | irritated | listless | down | nervous | bored | anxious | PA_mean | NA_mean |
| --- | --- | --- | --- | --- | --- | --- | --- | --- | --- | --- | --- | --- | --- | --- | --- |
| satisfied | ---- | 0.86 | 0.93 | 0.88 | 0.9 | 0.75 | -0.57 | -0.64 | -0.68 | -0.73 | -0.63 | -0.56 | -0.57 | 0.95 | -0.78 |
| relaxed |  | ---- | 0.83 | 0.81 | 0.76 | 0.9 | -0.61 | -0.67 | -0.58 | -0.68 | -0.75 | -0.47 | -0.62 | 0.91 | -0.76 |
| cheerful |  |  | ---- | 0.94 | 0.94 | 0.73 | -0.54 | -0.62 | -0.68 | -0.72 | -0.61 | -0.53 | -0.54 | 0.97 | -0.76 |
| energetic |  |  |  | ---- | 0.93 | 0.7 | -0.51 | -0.59 | -0.73 | -0.72 | -0.59 | -0.53 | -0.53 | 0.95 | -0.77 |
| enthusiastic |  |  |  |  | ---- | 0.65 | -0.47 | -0.55 | -0.66 | -0.7 | -0.54 | -0.49 | -0.49 | 0.94 | -0.71 |
| calm |  |  |  |  |  | ---- | -0.61 | -0.64 | -0.48 | -0.62 | -0.77 | -0.43 | -0.62 | 0.83 | -0.69 |
| upset |  |  |  |  |  |  | ---- | 0.68 | 0.53 | 0.7 | 0.71 | 0.6 | 0.7 | -0.58 | 0.74 |
| irritated |  |  |  |  |  |  |  | ---- | 0.6 | 0.7 | 0.72 | 0.63 | 0.66 | -0.66 | 0.83 |
| listless |  |  |  |  |  |  |  |  | ---- | 0.77 | 0.57 | 0.65 | 0.53 | -0.69 | 0.85 |
| down |  |  |  |  |  |  |  |  |  | ---- | 0.74 | 0.66 | 0.71 | -0.75 | 0.91 |
| nervous |  |  |  |  |  |  |  |  |  |  | ---- | 0.55 | 0.79 | -0.68 | 0.84 |
| bored |  |  |  |  |  |  |  |  |  |  |  | ---- | 0.54 | -0.54 | 0.74 |
| anxious |  |  |  |  |  |  |  |  |  |  |  |  | ---- | -0.59 | 0.76 |
| PA_mean |  |  |  |  |  |  |  |  |  |  |  |  |  | ---- | -0.80 |
| NA_mean |  |  |  |  |  |  |  |  |  |  |  |  |  |  | ---- |

Note: full description of the affect items (items 3-15) used are given in supplementary table S2. PA_mean = mean of person-means of the positive affect subscale. NA_mean = mean of person-means of the negative affect subscale. Details on calculation of the subscales are given in the Method section of the paper.

# S4 Explanation of within-person variance and autocorrelation, its correlation with RMSSD, and median (IQR) per group

In this supplement, we provide correlations of the root mean square successive difference (RMSSD) with other well-known variability measures within-person variance (WPV) and autocorrelation (see Table S4.1). We also report the median per group of the within-person variance (see Table S4.2) and autocorrelation (see Table S4.3) and the accompanying boxplots (see Figure S4.1 and S4.2). First, we explain how WPV and autocorrelation is calculated.

The RMSSD measures temporal instability, meaning that it takes both variability and temporal dependency into account (as explained in the main paper). These two effects can be separately studied with WPV (or standard deviation) and autocorrelation (or autocorrelation). WPV is a widely used measure of affective instability, because it is a familiar statistic. For *N* measurements, WPV is given by

$$\mathrm{WPV}=\frac{1}{N-1}\sum_{i=1}^{N} ({x_{i}-\bar{x})}^{2}$$

where $\bar{x}=\frac{\sum_{i=1}^{N} x_{i}}{N}.$ As follows from this equation, WPV does not take temporal dependency into account.

As opposed to WPV, autocorrelation does measure temporal dependency. autocorrelation measures temporal dependency between the (*i* + 1)th and the *i*th measurement of a time series. Assuming that measurements have equally spaced time intervals, we define the interval as *h*. The *h*th-order autocorrelation is given by

$Autocorrelation(h)=\frac{\sum_{i=1}^{N-h} {(x}_{i+h}-\bar{x})(x_{i}-\bar{x})}{\sum_{i=1}^{N} ({x_{i}-\bar{x})}^{2}}$.

The autocorrelation, thus, indicates how well measurements at time point *i* correlates with measurements at the previous time point. For a more thorough description of these measures and comparisons of RMSSD, WPV, and autocorrelation, see Jahng, Wood, and Trull (2008).

*Table S4.1. Correlation between root mean square successive difference (RMSSD), Within-person variance (WPV), and autocorrelation (inertia)* *of PA (above diagonal) and NA (below diagonal).*

|  | **RMSSD** | **WPV** | **Inertia** |
| --- | --- | --- | --- |
| **RMSSD** | --- | 0.92 | -0.02 |
| **WPV** | 0.97 | --- | 0.30 |
| **Inertia** | 0.37 | 0.55 | --- |

*Table S4.2: Median (IQR) of within-person variance (WPV) of positive (PA) and negative affect (NA) in diagnostic groups*

| **WPV** | **Current (*n* = 95)** | **Remitted (*n* = 178)** | **Control (*n* = 92)** | ***p***^a^ |
| --- | --- | --- | --- | --- |
| **PA** |  |  |  |  |
|  | 0.57 (0.29-0.83) | 0.39 (0.23-0.59) | 0.24 (0.13-0.40) | **<0.0001**^b^ |
| **NA** |  |  |  |  |
|  | 0.31 (0.18-0.48) | 0.12 (0.05-0.27) | 0.03 (0.02-0.07) | **<0.0001** ^b^ |

Note: ^a^ Kruskal–Wallis test; ^b^ Dunn’s test, *p* < 0.05 for all comparisons (Bonferroni corrected)

*Table S4.3: Median (IQR) auto-correlation of positive (PA) and negative affect (NA) in diagnostic groups.*

| **Inertia** | **Current (*n* = 95)** | **Remitted (*n* = 178)** | **Control (*n* = 92)** | ***p***^a^ |
| --- | --- | --- | --- | --- |
| **PA** |  |  |  |  |
|  | 0.25 (0.16-0.39) | 0.24 (0.14-0.38) | 0.17 (0.07-0.30) | **0.0017** ^b^ |
| **NA** |  |  |  |  |
|  | 0.30 (0.17-0.42) | 0.20 (0.08-0.32) | 0.05 (-0.04-0.21) | **<0.0001** ^c^ |

Note: ^a^ Kruskal–Wallis test; ^b^ Dunn’s test, *p* < 0.05 for all comparisons except Current vs Remitted (Bonferroni corrected); ^c^ Dunn’s test, *p* < 0.0001 for all three comparisons (Bonferroni corrected)


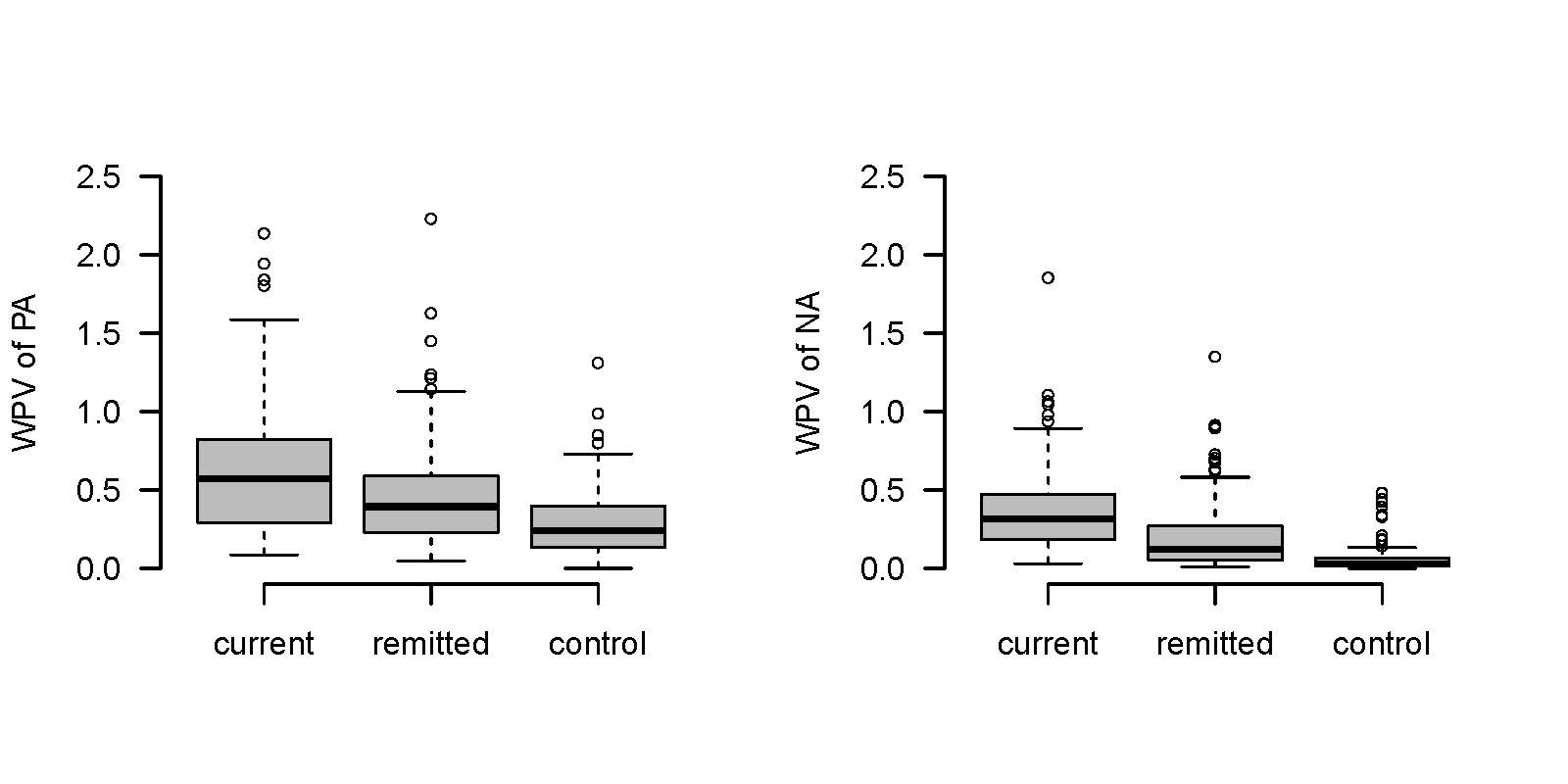


Figure S4.1 Boxplots of within-person variance of PA and NA of the diagnostic groups


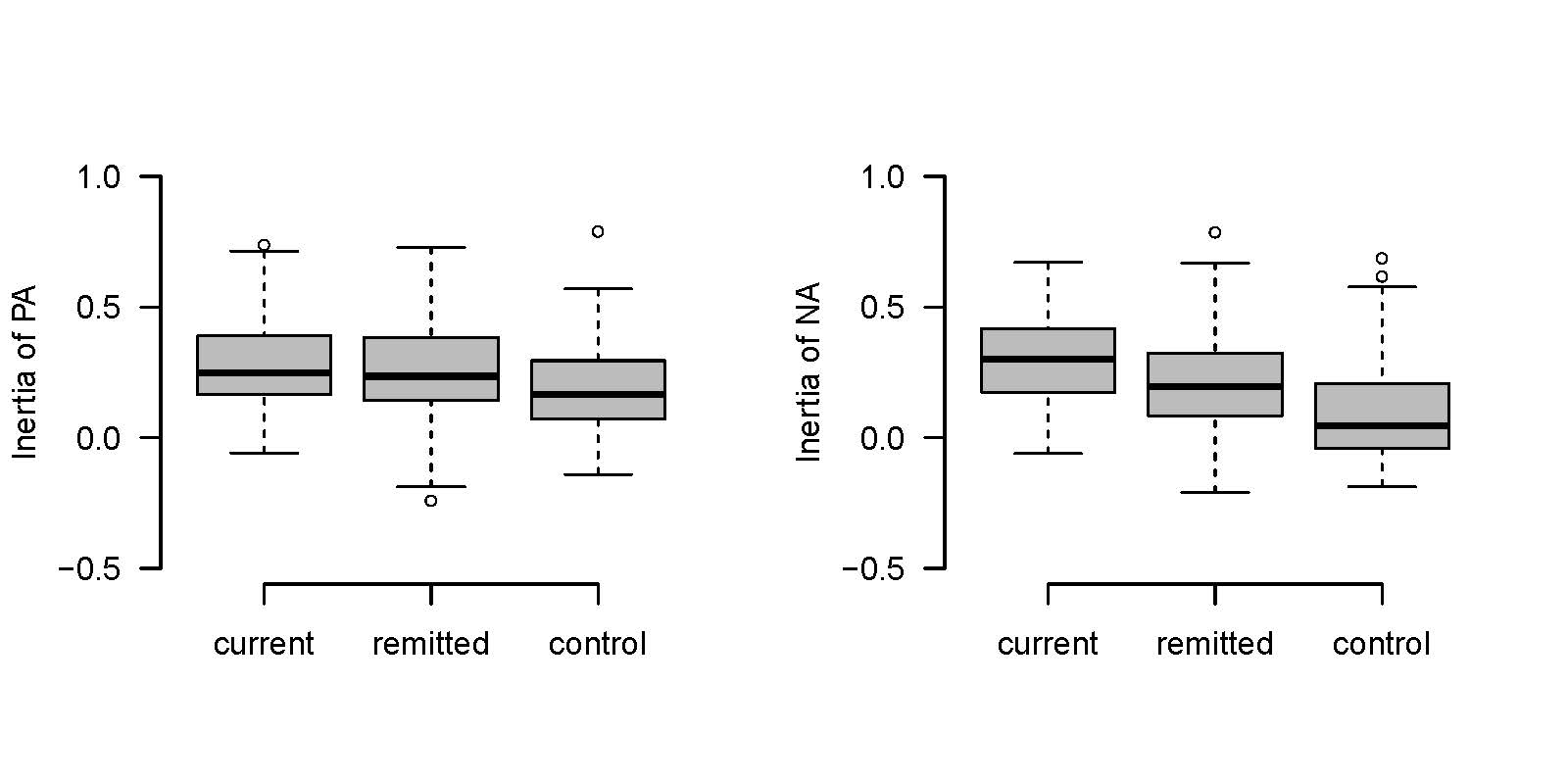
Figure S4.2 Boxplots of the auto-correlation (inertia) of PA and NA of the diagnostic groups

# S5 Median (IQR) of the corrected RMSSD (cRMSSD) of positive and negative affect in diagnostic groups

| **cRMSSD** | **Current (*n* = 95)** | **Remitted (*n* = 178)** | **Control (*n* = 92)** | ***p***^a^ | ***ε*^2^** |
| --- | --- | --- | --- | --- | --- |
| **PA** |  |  |  |  |  |
|  | 0.19 (0.15-0.26) | 0.15 (0.12-0.21) | 0.11 (0.08-0.15) | **<0.0001** ^b^ | 0.19 |
| **NA** |  |  |  |  |  |
|  | 0.32 (0.23-0.40) | 0.30 (0.22-0.37) | 0.20 (0.16-0.27) | **<0.0001** ^c^ | 0.18 |

Note: ^a^ Kruskal–Wallis test; ^b^ Dunn’s test, *p* < 0.0001 for all three comparisons (Bonferroni corrected); ^c^ Dunn’s test, *p* < 0.0001 except for Current vs Remitted (Bonferroni corrected); *ε*^2^, effect size

# S6 Median (IQR) RMSSD of positive and negative affect in diagnostic groups after excluding respondents with pure current or pure remitted anxiety disorder

| **RMSSD** | **Current (*n* = 88)** | **Remitted (*n* = 153)** | **Control (*n* = 92)** | ***p***^a^ | ***ε*^2^** |
| --- | --- | --- | --- | --- | --- |
| **PA** |  |  |  |  |  |
|  | 0.80 (0.64-1.06) | 0.78 (0.61-0.90) | 0.61 (0.44-0.78) | **<0.0001** ^b^ | 0.10 |
| **NA** |  |  |  |  |  |
|  | 0.63 (0.47) | 0.43 (0.31-0.63) | 0.24 (0.17-0.32) | **<0.0001** ^c^ | 0.34 |

Note: ^a^ Kruskal–Wallis test; ^b^ Dunn’s test, *p* < 0.05 except for Current vs Remitted, (Bonferroni corrected); ^c^ Dunn’s test, *p* < 0.05 for all three comparisons, Bonferroni corrected; *ε*^2^, effect size

References

Jahng, S., Wood, P. K., & Trull, T. J. (2008). Analysis of affective instability in ecological momentary assessment: Indices using successive difference and group comparison via multilevel modeling. *Psychological Methods, 13*(4), 354-375. doi:10.1037/a0014173 [doi]

Servaas, M. N., Riese, H., Renken, R. J., Wichers, M., Bastiaansen, J. A., Figueroa, C. A., . . . Ruhe, H. G. (2017). Associations between daily affective instability and connectomics in functional subnetworks in remitted patients with recurrent major depressive disorder. *Neuropsychopharmacology: Official Publication of the American College of Neuropsychopharmacology, 42*(13), 2583-2592. doi:10.1038/npp.2017.65 [doi]
